# Comprehensive immunohistochemical study of mesothelin (MSLN) using different monoclonal antibodies 5B2 and MN-1 in 1562 tumors with evaluation of its prognostic value in malignant pleural mesothelioma

**DOI:** 10.18632/oncotarget.15814

**Published:** 2017-03-01

**Authors:** Shingo Inaguma, Zengfeng Wang, Jerzy Lasota, Masanori Onda, Piotr Czapiewski, Renata Langfort, Janusz Rys, Joanna Szpor, Piotr Waloszczyk, Krzysztof Okoń, Wojciech Biernat, Hiroshi Ikeda, David S. Schrump, Raffit Hassan, Ira Pastan, Markku Miettinen

**Affiliations:** ^1^ Laboratory of Pathology, National Cancer Institute, Bethesda, MD, USA; ^2^ Department of Pathology, Aichi Medical University School of Medicine, Nagakute, Japan; ^3^ Laboratory of Molecular Biology, National Cancer Institute, Bethesda, MD, USA; ^4^ Department of Pathomorphology, Medical University of Gdansk, Gdansk, Poland; ^5^ Department of Pathology, Otto-von-Guericke University Magdeburg, Magdeburg, Germany; ^6^ Department of Pathology, National Tuberculosis and Lung Diseases Research Institute, Warsaw, Poland; ^7^ Department of Tumor Pathology, Centre of Oncology, Maria Sklodowska-Curie Memorial Institute, Krakow Branch, Poland; ^8^ Department of Pathomorphology, Jagiellonian University, Krakow, Poland; ^9^ Independent Laboratory of Pathology, Zdunomed, Szczecin, Poland; ^10^ Thoracic and GI Oncology Branch, National Cancer Institute, Bethesda, MD, USA

**Keywords:** mesothelin (MSLN), immunohistochemistry, 5B2, MN-1, malignant pleural mesothelioma

## Abstract

Mesothelin (MSLN) is a glycophosphatidylinositol (GPI)-linked cell surface protein highly expressed in several types of malignant tumors sometimes in association with increased tumor aggressiveness and poor clinical outcome. In the present study, 1562 tumors were immunohistochemically analyzed for mesothelin expression using two different types of mouse monoclonal antibodies (5B2 and MN-1) to determine the clinical usefulness of mesothelin immunohistochemistry as well as to pinpoint potential targets for future anti-mesothelin therapy. Also, characterization of selected mesothelin-positive tumors was performed by immunohistochemistry and oncogene sequencing. Among the tumors analyzed, the highest frequencies of mesothelin-positivity were detected in ovarian serous carcinoma (90% in 5B2 and 94% in MN-1). Both antibodies showed frequent positivity in pancreatic adenocarcinoma (71% using 5B2 and 87% using MN-1) and malignant pleural mesothelioma (75% using 5B2 and 78% using MN-1). In malignant mesothelioma, overall survival was significantly longer in the cohort of patients with diffuse membranous expression of mesothelin (*P* < 0.001). Both antibodies showed positive staining in thymic carcinoma (77% in 5B2 and 59% in MN-1), however, no expression was detected in thymoma. No correlation was detected between mesothelin expression and mismatch repair system deficient phenotype or gene mutation (*BRAF* and *RAS*) status in gastrointestinal adenocarcinomas. Mesothelin immunohistochemistry may assist the differential diagnosis of thymoma vs. thymic carcinoma as well as prognostication of mesothelioma patients. Our results demonstrate that patients with solid tumors expressing mesothelin could be targeted by anti-mesothelin therapies.

## INTRODUCTION

Mesothelin (MSLN) is a glycophosphatidylinositol (GPI)-linked cell surface protein normally expressed in mesothelial cells that line the pleura, peritoneum, and pericardium. The *MSLN* gene encodes a precursor protein of 71 kDa that is processed to a 31 kDa shed protein called MPF (megakaryocyte potentiating factor) and a 40 kDa membrane bound protein, mesothelin [1]. The biologic function of mesothelin is not well known, however, no detectable abnormalities were reported in growth and reproduction in a *mesothelin* deficient mouse model [2].

Mesothelin is reported to be highly expressed in several types of malignant tumors, such as malignant mesothelioma, ovarian cancer, pancreatic adenocarcinoma, and lung adenocarcinoma. In some cases, mesothelin expression has been associated with increased tumor aggressiveness and poor clinical outcome, however, its impact on the clinical outcome of malignant pleural mesothelioma patients has not been extensively evaluated [3–10]. In ovarian cancer, it has been shown that mesothelin binds to ovarian cancer antigen MUC16 (CA-125) and may contribute to dissemination into the abdominal cavity [11–13]. It has also been shown that mesothelin plays a pivotal role in tumor cell proliferation, invasion, and chemotherapy resistance through the activation of oncogenic signaling [14–16]. Although the mechanism(s) and/or tumor biological significances were unclear, high mesothelin expression was associated with *KRAS* gene mutation in lung adenocarcinoma [8, 9].

Anti-mesothelin immunotherapies for mesothelin-expressing tumor include use of recombinant immunotoxin (SS1P), a high-affinity chimeric monoclonal antibody (MORAb-009), an anti-mesothelin antibody drug conjugate (BAY 94-9343), and adoptive T-cell immunotherapy using mesothelin-specific chimeric antigen receptors (CAR) [17]. Serum mesothelin levels have been found to correlate with mesothelioma responsiveness to anti-mesothelin therapies [18–21], however, it has not been shown whether immunohistochemistry can be used as a biomarker to predict clinical response to these drugs.

The aim of this study was to evaluate differential reactivity of different types of mouse monoclonal antibodies against mesothelin as well as MPF/precursor mesothelin for immunohistochemistry. It was also aimed to determine the clinical usefulness of mesothelin immunohistochemistry as well as to highlight tumor types for future mesothelin-targeting therapy. Additional immunohistochemical and oncogene mutation analyses were performed to characterize the mesothelin-positive tumors.

## RESULTS

### Comparison of two mesothelin and three MPF antibodies in 218 selected tumor tissues

**Figure 1:**
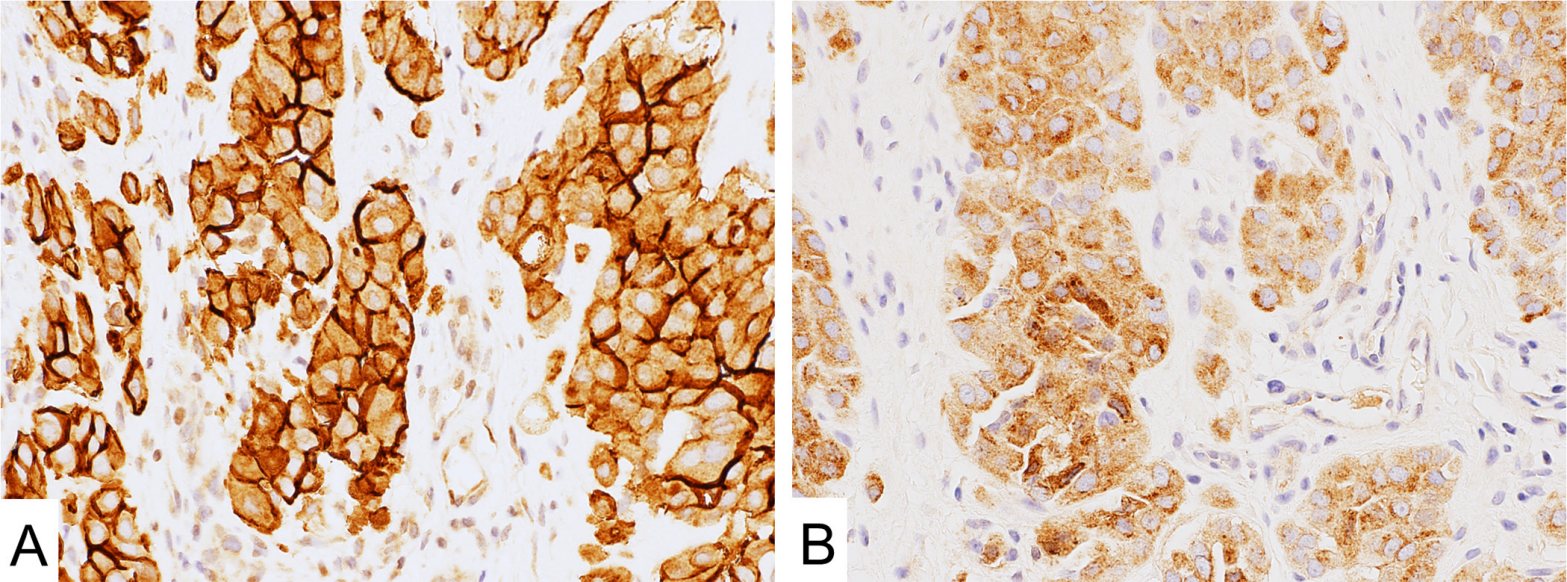
Mesothelin expression in malignant mesothelioma (**A**) MN-1 antibody showed strong signal on the cell membrane. (**B**) MPF44 antibody showed cytoplasm-dominant staining.

Immunohistochemical staining using two mesothelin (5B2, and MN-1) and three MPF (MPF25, MPF44, and MPF49) antibodies were performed in 218 selected tumor tissues including ovarian serous carcinoma, pancreatic ductal carcinoma, thymic tumors, and malignant mesothelioma. Among them, MPF49 antibody did not generate sufficient specific staining signals in a selection of mesothelin-positive tumors and was not studied further (data not shown). The results of immunohistochemistry using the two anti-mesothelin and two anti-MPF antibodies have been summarized in Table 1. The mesothelin antibodies (5B2 and MN-1) showed higher rates of positivity than MPF antibodies (MPF 25 and MPF 44) in all of the tumors analyzed. In malignant mesothelioma, mesothelin antibodies (5B2 and MN-1) showed membrane positivity, whereas MPF44 showed predominantly cytoplasmic staining. (Figure 1A and 1B) From this preliminary experiment, 2 mesothelin antibodies, 5B2 and MN-1, were chosen for the study of normal tissues and a larger cohort of tumors because of their higher rates of positivity on initial screening.

**Table 1:** Mesothelin expression in different types of tumors detected by clone 5B2, MN-1, MPF44 and MPF25 antibodies

Diagnosis		Total	5B2						MN-1						MPF44						MPF25					
		No.	Luminal/membrane			Total*			Luminal/membrane			Total*			Luminal/membrane			Total*			Luminal/membrane			Total*		
			No.	%	Range (median)	No.	%	Range (median)	No.	%	Range (median)	No.	%	Range (median)	No.	%	Range (median)	No.	%	Range (median)	No.	%	Range (median)	No.	%	Range (median)
Tumors with adenocarcinoma-like differentiation																										
	Ovary, serous carcinoma	47	42	89.4	5–100 (93)	44	93.6	5–100 (90)	44	93.6	5–100 (100)	44	93.6	5–100 (100)	34	72.3	5–100 (60)	40	85.1	5–100 (80)	14	29.8	5–60 (30)	25	53.2	5–100 (40)
	Pancreas, invasive ductal carcinoma	47	28	59.6	10–90 (60)	30	63.8	10–100 (80)	40	85.1	10–100 (75)	42	89.4	10–100 (100)	33	70.2	10–100 (60)	41	87.2	5–100 (90)	18	38.3	10–90 (35)	32	68.1	10–100 (80)
Tumors with squamous cell differentiation																										
	Thymic carcinoma	17	6	35.3	5–100 (93)	13	76.5	10–100 (100)	7	41.2	5–100 (90)	10	58.8	10–100 (95)	3	17.6	10–100 (10)	7	41.2	10–100 (70)	1	5.9	20 (20)	3	17.6	5–100 (10)
	Thymoma	33	0	0.0	-	0	0.0	-	0	0.0	-	0	0.0	-	0	0.0	-	0	0.0	-	0	0.0	-	0	0.0	-
Other epithelial tumors																										
	Malignant mesothelioma	28	24	85.7	5–100 (80)	25	89.3	5–100 (100)	23	82.1	10–100 (80)	25	89.3	10–100 (100)	8	28.6	10–60 (35)	20	71.4	10–100 (100)	1	3.6	40 (40)	9	32.1	10–100 (80)
	Urinary tract, urothelial carcinoma	46	6	13.0	5–50 (15)	11	23.9	10–100 (60)	5	10.9	5–50 (40)	10	21.7	5–95 (45)	0	0.0	-	4	8.7	5–50 (30)	0	0.0	-	0	0.0	-

MPF49 antibody was eliminated from our study because of its highest back ground signals with no specific staining. *, total indicates mesothelin expression on luminal/membrane and/or cytoplasm.

### Immunohistochemistry using 5B2 and MN-1 antibodies in normal tissues and 1562 tumors

**Figure 2:**
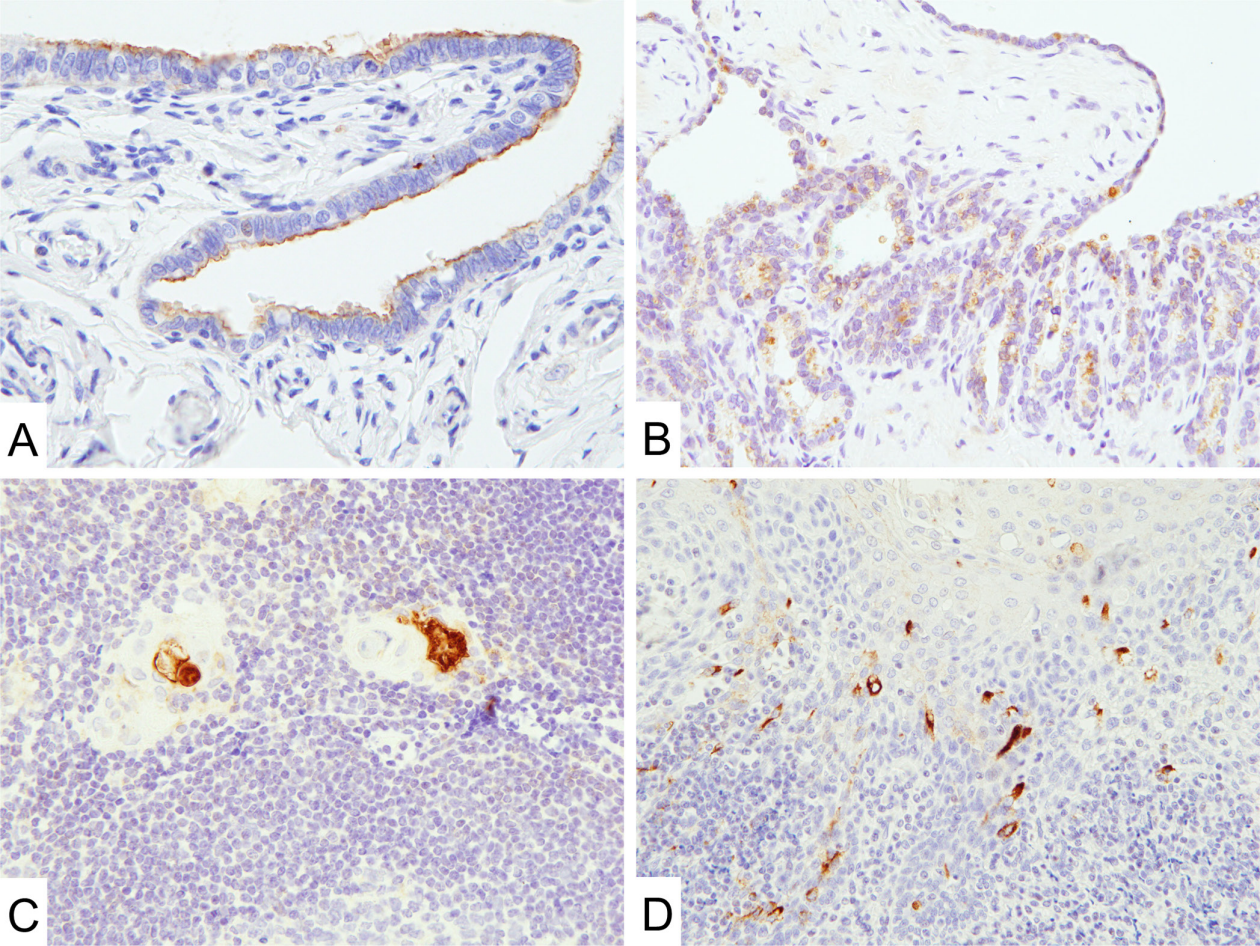
Mesothelin expression in normal tissues (**A** and **B**) MN-1 antibodies showed limited signal in the epithelium of fallopian tubes (**A**) and seminal vesicles (**B**). (**C** and **D**) Hassall's corpuscles of the thymus (**C**) and a subset of squamous epithelial cells of the tonsils (**D**) showed mesothelin expression.

In normal tissues, both 5B2 and MN-1 antibodies showed limited mesothelin expression in the epithelium of fallopian tubes and seminal vesicles (Figure 2A and 2B). Also, Hassall's corpuscles of the thymus and a subset of squamous epithelial cells of the tonsils showed mesothelin expression (Figure 2C and 2D).

**Figure 3:**
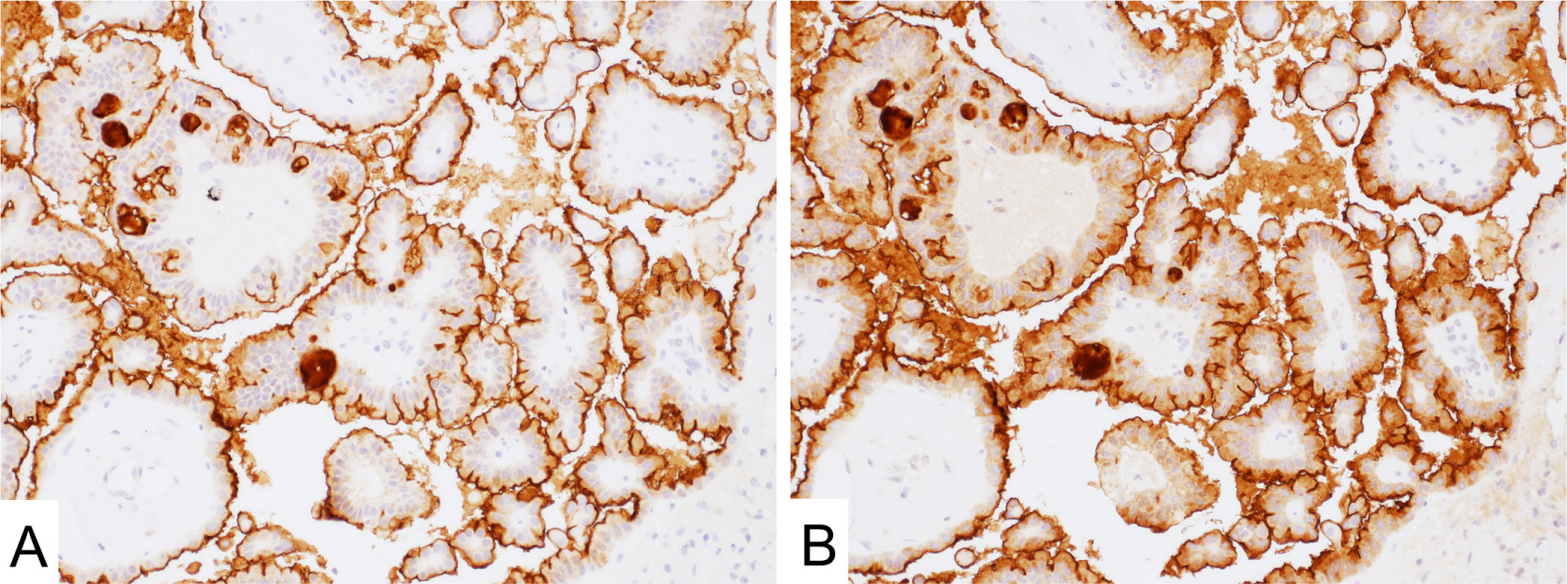
Mesothelin expression in ovary serous carcinoma (**A** and **B**) Both 5B2 (**A**) and MN-1 (**B**) antibodies showed strong signal on the apical side of the cell membrane.

Table 2 summarizes the results of immuno histochemistry for mesothelin using mouse monoclonal antibodies 5B2 and MN-1 in different types of tumors. Among them, high frequencies of mesothelin-positivity were detected in ovarian serous carcinoma (90%, median value of positive cells 90% in 5B2 and 94%, median value of positive cells 100% in MN-1) (Figure 3A and 3B).

**Table 2:** Mesothelin expression in different types of tumors detected by clone 5B2 and MN-1 antibodies

	Diagnosis		Total	Mesothelin (5B2)						Mesothelin (MN-1)					
			No.	Luminal/membrane			Total*			Luminal/membrane			Total*		
				No.	%	Range (median)	No.	%	Range (median)	No.	%	Range (median)	No.	%	Range (median)
Tumors with adenocarcinoma-like differentiation															
	Ovary, serous carcinoma		79	71	89.9	5–100 (90)	73	92.4	5–100 (90)	74	93.7	5–100 (100)	75	94.9	5–100 (100)
	Pancreas, invasive ductal carcinoma		132	93	70.5	5–100 (50)	100	75.8	5–100 (80)	115	87.1	5–100 (80)	119	90.2	10–100 (100)
	Uterus, endometrioid adenocarcinoma		82	52	63.4	5–100 (20)	62	75.6	5–100 (30)	51	62.2	5–100 (30)	59	72.0	5–100 (30)
	Colorectum, adenocarcinoma		188	91	48.4	5–100 (10)	108	57.4	5–100 (30)	115	61.2	5–100 (30)	128	68.1	5–100 (45)
	Lung, adenocarcinoma		76	36	47.4	5–100 (30)	50	65.8	5–100 (80)	31	40.8	5–100 (60)	47	61.8	5–100 (100)
	Liver, cholangiocellular carcinoma		39	16	41.0	10–100 (60)	16	41.0	20–100 (95)	16	41.0	10–90 (80)	16	41.0	30–100 (100)
	Stomach, adenocarcinoma		81	31	38.3	5–90 (20)	60	74.1	5–100 (80)	40	49.4	5–100 (20)	63	77.8	5–100 (90)
	Mammary gland, invasive ductal carcinoma		119	13	10.9	5–60 (10)	13	10.9	5–100 (60)	14	11.8	5–80 (20)	16	13.4	5–100 (75)
	Prostate gland, adenocarcinoma		107	0	0.0	-	1	0.9	90 (−)	0	0.0	-	1	0.9	90 (−)
	Mammary gland, lobular carcinoma		82	0	0.0	-	0	0.0	-	0	0.0	-	4	4.9	5–100 (70)
Tumors with squamous cell differentiation															
	Thymic carcinoma		17	6	35.3	5–100 (93)	13	76.5	10–100 (100)	7	41.2	5–100 (90)	10	58.8	10–100 (95)
	Lung, squamous cell carcinoma		72	20	27.8	5–90 (10)	33	45.8	5–100 (20)	11	15.2	5–100 (20)	20	27.8	5–100 (20)
	Uterine cervix, squamous cell carcinoma		21	3	14.3	20–40 (20)	6	28.6	20–100 (90)	3	14.3	20–40 (20)	6	28.6	20–100 (90)
	Thymoma		33	0	0.0	-	0	0.0	-	0	0.0	-	0	0.0	-
Other epithelial tumors															
	Malignant mesothelioma		143	107	75.0	5–100 (90)	111	77.6	5–100 (100)	111	77.6	5–100 (100)	113	79.0	10–100 (100)
	Epithelioid		115	93	80.9	5–100 (90)	97	84.3	5–100 (100)	97	84.3	5–100 (100)	99	86.1	10–100 (100)
	Biphasic		17	11	64.7	20–100 (70)	11	64.7	20–100 (100)	11	64.7	30–100 (80)	11	64.7	30–100 (100)
	Sarcomatoid		10	2	20.0	80 (80)	2	20.0	90–100 (95)	2	20.0	40–80 (60)	2	20.0	90–100 (95)
	Urinary tract, urothelial carcinoma		85	11	12.9	5–100 (20)	18	21.2	10–100 (35)	7	8.2	5–100 (40)	14	16.5	5–100 (40)
	Kidney, clear cell renal cell carcinoma		206	9	4.4	5–100 (20)	15	7.3	5–100 (50)	17	8.3	5–100 (20)	24	11.7	5–100 (30)

* total indicates mesothelin expression on luminal/membrane and/or cytoplasm.

**Figure 4:**
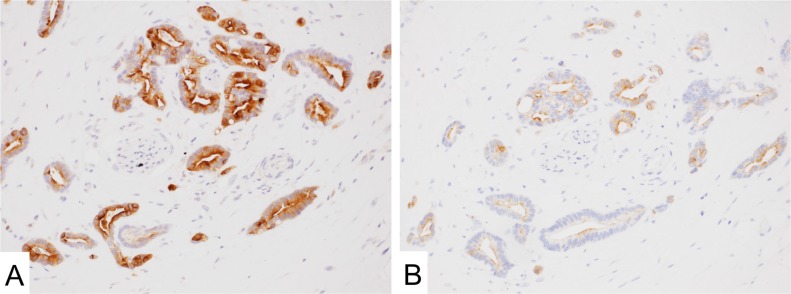
Mesothelin expression in adenocarcinoma of the pancreas (**A** and **B**) MN-1 (**A**) showed higher positivity than 5B2 (**B**) antibody.

In other tumors with adenocarcinoma-like differentiation, both clones showed variable positivity on the cell membrane and cytoplasm of the neoplastic cells. MN-1 showed higher positive staining rate than 5B2 on the cell membrane in invasive ductal carcinoma of the pancreas (87% vs. 71%, Figure 4A and 4B), adenocarcinomas of the colorectum (61% vs. 48%) and stomach (49% vs. 38%).

Both antibodies showed similar positivity in malignant mesothelioma (75% in 5B2 and 78% in MN-1), uterine endometrioid adenocarcinoma (63% vs. 62%), intrahepatic cholangiocellular carcinoma (41% each), and invasive ductal carcinoma of the mammary gland (11% vs. 12%). In malignant mesothelioma, 31% (45/143) and 40% (57/143) of cases showed diffuse mesothelin expression with 5B2 and MN-1 immunostaining, respectively. Epithelioid mesothelioma showed significantly higher mesothelin expression (81% with 5B2 and 84% with MN-1) than sarcomatoid (20% with 5B2 and 20% with MN-1) and biphasic (65% with 5B2 and 65% with MN-1) histotypes with both antibodies.

In adenocarcinoma of the lung, 5B2 showed slightly higher positivity (47%) than MN-1 (41%).

Adenocarcinoma of the prostate and lobular carcinoma of the breast were usually negative but occasionally showed cytoplasmic mesothelin-positivity (1–5% of cases).

**Figure 5:**
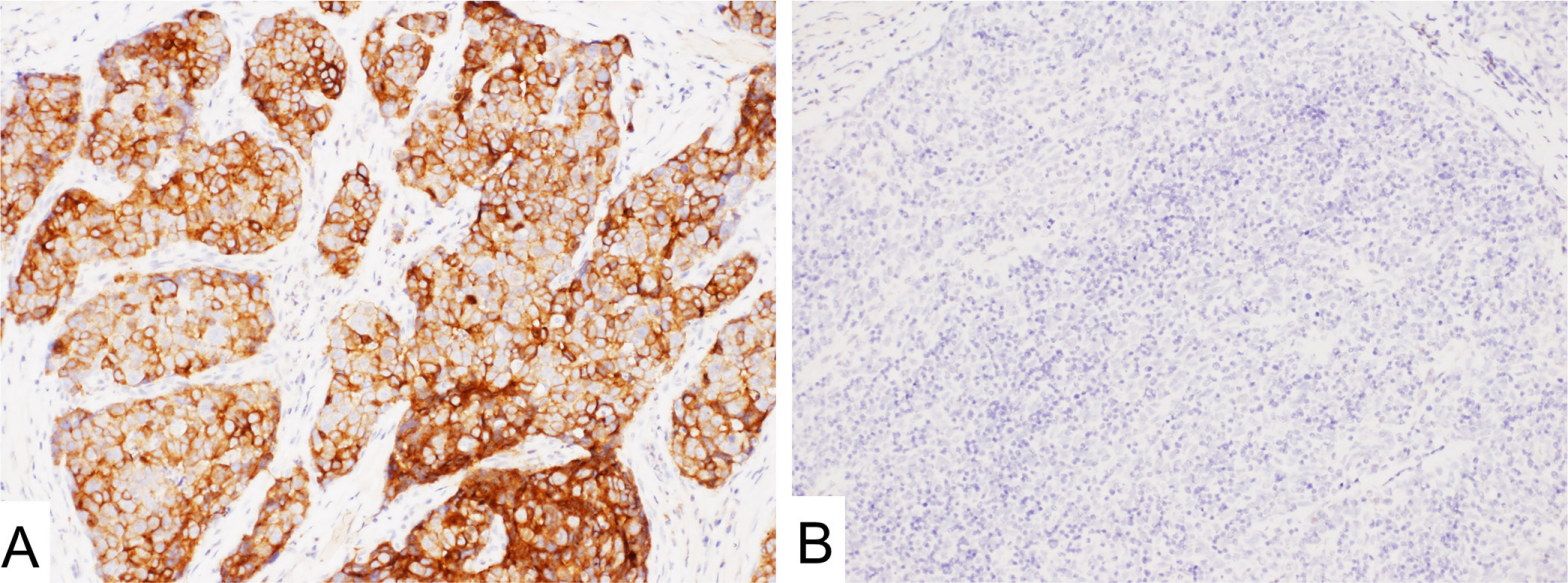
Mesothelin immunostaining with 5B2 in thymic tumors (**A** and **B**) thymic carcinoma (**A**) showed diffuse cytoplasm-dominant staining, however, thymoma (**B**) showed no signal.

Forty-one percent of thymic carcinomas were positive for mesothelin on the cell membrane with MN-1 antibody. 5B2 showed dominant cytoplasmic staining in these tumors (Figure 5A). On the other hand, benign thymomas showed no positivity with either antibody (Figure 5B).

Squamous cell carcinoma of the lung (28% with 5B2, 15% with MN-1) and uterine cervix (14% with 5B2, 14% with MN-1) showed lower positivity than thymic carcinoma in this study.

**Figure 6:**
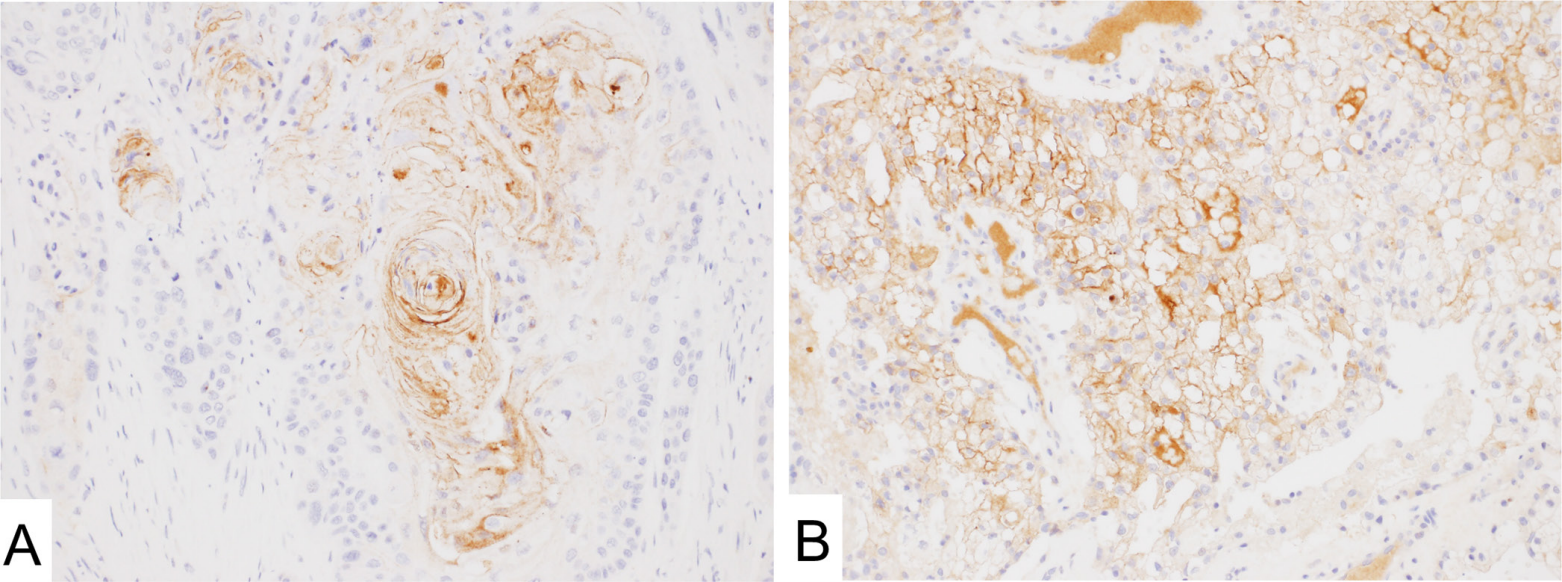
Mesothelin immunostaining with 5B2 in urothelial and clear cell renal cell carcinoma (**A** and **B**) urothelial carcinoma (**A**) showed positive staining on the site of squamous metaplastic differentiation. Clear cell renal cell carcinoma (**B**) showed positive signal on the cell membrane.

Urothelial carcinomas and clear cell renal cell carcinomas showed rare positivity for mesothelin with both antibodies (4–13%, Figure 6A and 6B). In urothelial carcinoma, positive signals were especially detected in components with squamous cell differentiation (Figure 6A).

### Survival analysis of mesothelioma patients

**Figure 7:**
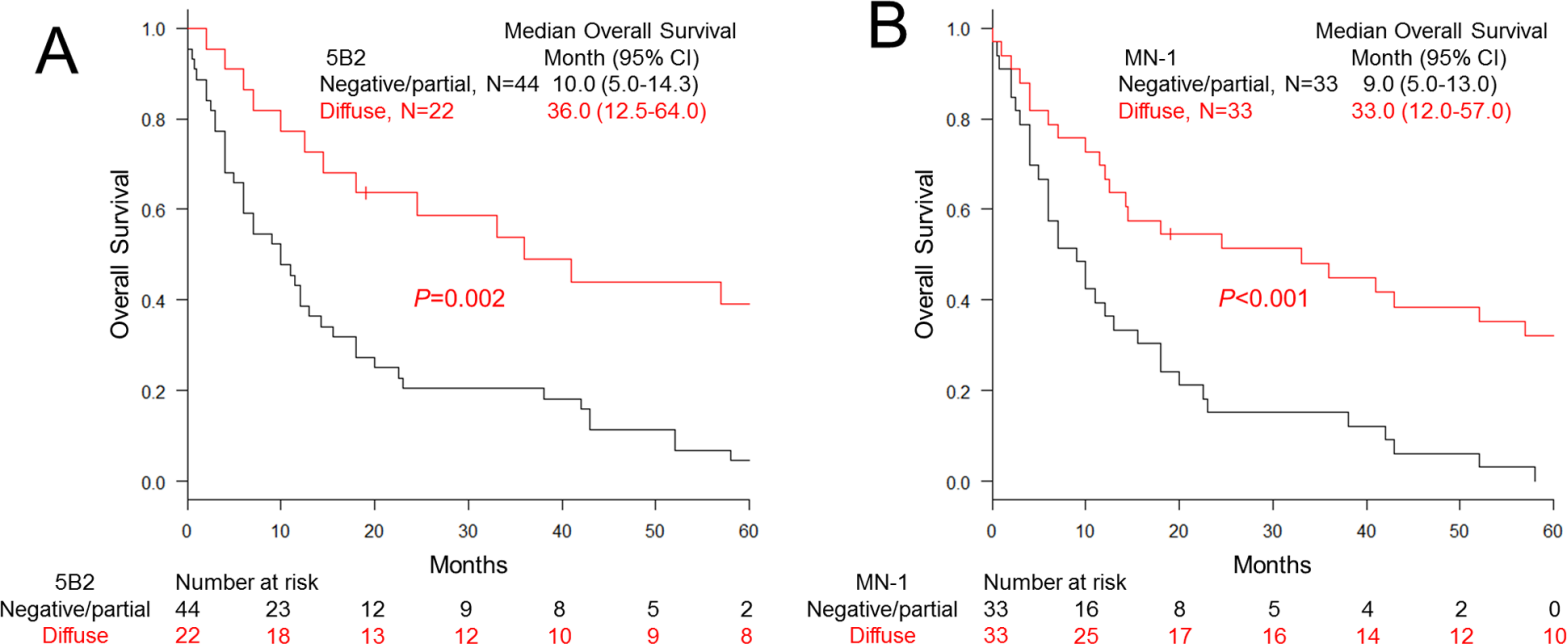
Overall survival of malignant pleural mesothelioma cases classified with mesothelin expression (**A** and **B**) Kaplan-Meier curves for the patients carrying mesothelioma grouped with diffuse membranous or partial/negative mesothelin expression stained with 5B2 (**A**) and MN-1 (**B**) antibodies.

Characteristics of the 66 mesothelioma patients analyzed for survival have been summarized in Table 3. Patients were followed up for up to 120 months. Survival was significantly longer for patients with mesothelioma expressing mesothelin diffusely (100% of positive cells) on the cell membrane using 5B2 (36.0 months median vs. 10.0 months with heterogeneous or no mesothelin expression; *P* = 0.002, Figure 7A) or MN-1 (33.0 months median vs. 9.0 months with heterogeneous or no mesothelin expression; *P* < 0.001, Figure 7B) antibodies. Multivariable Cox hazards regression analysis revealed diffuse membranous mesothelin expression in mesothelioma tumor cells to be a favorable prognostic factor (HR, 0.36; 95% CI, 0.21–0.64; *P* < 0.001) (Table 4).

**Table 3:** Characteristics of the 66 mesothelioma patients analyzed for survival

Characteristics		
Age, year		
Mean	60.9 ± 10.5	
Median (range)	61	(27–87)
Sex, no. (%)		
Male	50	(76)
Female	16	(24)
Histology, no. (%)		
Epithelial	56	(85)
Biphasic	7	(11)
Sarcomatoid	3	(5)
Tumor with diffuse membranous mesotheline (MN-1), no. (%)		
	33	(50)

**Table 4:** Cox hazard ratio of malignant pleural mesothelioma patients

	Hazard	95% CI			
	Ratio	min	max	*P*-value	
Membranous mesothelin expression (MN-1)					
Partial or negative	1				
Diffuse (100% of positive cells on the cell membrane)	0.36	0.21	0.64	< 0.001	*

The multivariable Cox hazards analysis model initially included age, sex, tumor histology, and mesothelin expression (MN-1 antibody).

A backward elimination with a threshold of *P* = 0.05 was used to select variables in the final model.

### Correlation with mismatch repair system deficiency in gastrointestinal adenocarcinomas

In gastrointestinal adenocarcinomas, 16% (29/183) of colorectal and 12% (9/77) of gastric adenocarcinomas showed mismatch repair (MMR)-deficient phenotypes. No significant correlation was detected between MMR-deficiency and mesothelin-expression in gastric (*P* = 0.08) and colorectal (*P* = 0.51) adenocarcinomas (Supplementary Tables 1 and 2).

### Correlation with gene mutation status in colorectal adenocarcinomas

Arbitrary selected 75 colorectal tumors were analyzed for *BRAF*, *KRAS*, and *NRAS* gene mutations. Among them, 20 tumors showed BRAF V600E mutation and 30 tumors carried a mutated *KRAS* gene. Only one case showed mutation in the *NRAS* gene. All of the gene mutations were detected in a mutually exclusive manner. No statistical correlation was detected between mesothelin expression and gene mutation status (*P* = 0.82, Supplementary Table 3).

## DICUSSION

Mesothelin (MSLN) is a cell surface protein highly expressed in several types of malignant tumors, such as malignant mesothelioma, ovarian cancer, pancreatic adenocarcinoma, and lung adenocarcinoma, sometimes in association with increased tumor aggressiveness and poor clinical outcome. Several antibody-based therapeutic agents, vaccines, and T cell therapies against mesothelin, in some case, have shown favorable results [17]. Serum mesothelin levels have been found to correlate with mesothelioma responsiveness to anti-mesothelin therapies [12, 13, 18, 19]. However, the clinical usefulness of mesothelin immunohistochemistry, including for the prognostication of malignant pleural mesothelioma patients, has not been fully examined [3–10].

In the present study, mesothelin expression was evaluated using five different mouse monoclonal antibodies against mesothelin (clone 5B2 and MN-1) and MPF (megakaryocyte potentiating factor)/precursor mesothelin (MPF25, MPF44 and MPF49), in order to determine the optimal antibodies for mesothelin detection in common carcinomas. Since the anti-mesothelin (5B2 and MN-1) antibodies offered a superior signal quality in a preliminary study, they were used to analyze 1562 tumors to compare immunoreactivities of selected tumors as well as to test their clinical utility for pathological diagnosis and prognostication of mesothelioma patients. Furthermore, immunohistochemistry (MLH1, MSH2, MSH6, and PMS2) and gene mutation analyses (*BRAF*, *KRAS*, and *NRAS*) were performed to characterize the mesothelin-positive tumors.

Almost all ovarian serous carcinomas showed high-levels of mesothelin expression with both 5B2 and MN-1 anti-mesothelin antibodies. In the present study, these antibodies also showed similar high positivity (up to 70–80% of cases) in malignant mesothelioma and uterine endometrioid adenocarcinoma. Patients with these mesothelin-positive tumors are potential candidates for the mesothelin-targeting therapeutics such as a recombinant immunotoxin (SS1P), a high-affinity chimeric monoclonal antibody (MORAb-009), an anti-mesothelin antibody drug conjugate (BAY 94-9343), or adoptive T-cell immunotherapy using mesothelin-specific chimeric antigen receptors (CAR) in autologous T-lymphocytes [15].

Up to 80% of thymic carcinomas showed homogeneous and strong cell membrane and/or cytoplasmic staining for mesothelin. On the other hand, thymomas showed no positivity for mesothelin immunostaining with either antibody. Therefore, mesothelin immunohistochemistry with either antibody could have a role in differentiating thymic carcinomas from thymomas.

In adenocarcinomas arising in the pancreas, stomach, and colorectum, MN-1 antibodies showed a more prominent mesothelin positivity rate than 5B2. On the other hand, squamous cell carcinoma of the lung and urothelial carcinoma of the urinary tract, 5B2 showed slightly higher positivity.

In ELISA and immunoblot assay, MN-1 antibody showed much higher affinity than 5B2 antibody. Furthermore, 5B2 antibody showed lower or no affinity to native mesothelin expressed in human cells [22]. However, appropriate antigen retrieval and dilution of the antibodies led to favorable immunohistochemical staining results with 5B2 antibody [22]. It is likely that the different staining patterns and rates of positivity obtained with 5B2 and MN-1 antibodies are due to the differential expression of the various mesothelin epitopes in these tumors. Furthermore, these results suggest that appropriate type of anti-mesothelin antibody should be used when evaluating mesothelin expression in different tumor types. A thorough analysis of mesothelin expression in tumors being targeted by anti-mesothelin agents will be important to understand the correlation between mesothelin expression and treatment response.

Among the MPF antibodies, MPF44 has been shown to react with the full-length mesothelin precursor protein, a 71 kDa protein without cleavage, and the shed 31 kDa MPF, in immunoblot analyses [23]. These results indicate that mesothelin precursor protein might also be detected in immunohistochemistry by MPF25 and MPF 44 antibodies, however, soluble factor MPF may be harder to detect in formalin-fixed paraffin-embedded (FFPE) tissue sections. Our results suggest that malignant mesotheliomas express processed mesothelin on the cell membrane because 5B2 and MN-1 typically showed membrane positivity, whereas MPF44 showed predominantly cytoplasmic staining. We also show that MPF49 does not react with MPF protein in immunoblot analysis under SDS-denatured conditions probably indicating reactivity with a conformation-sensitive epitope [23]. This might also explain why MPF49 showed no specific staining in FFPE sections in this study.

In the present study, different from other types of tumors such as adenocarcinoma of the lung and stomach, mesothelin expression itself did not show any impact on the survival of the mesothelioma patients (data not shown) [8–10]. However, patients with malignant mesothelioma expressing mesothelin diffusely (100% of positive cells on the cell membrane) had increased overall survival compared to patients whose tumors had heterogeneous or no mesothelin expression. Since we did not have detailed clinical information about other patient characteristics such as clinical stage of their disease or performance status it is difficult to say whether diffuse mesothelin expression by itself is an independent favorable prognostic factor. This will need to be validated in larger studies with defined patient characteristics.

Several types of lung carcinomas have been reported to express mesothelin [6]. In case of lung adenocarcinoma high-level of mesothelin expression is associated with aggressiveness, poor prognosis and *KRAS* gene mutation status [8, 9]. Based on these observations, in the present study, gene mutation analyses were performed in colorectal adenocarcinomas since they commonly harbor *KRAS* mutations. Among 75 arbitrarily selected colorectal adenocarcinomas, 20 and 31 tumors carried *BRAF* and *RAS* mutations, respectively. However, no significant correlation between mesothelin-expression and gene mutation status was detected. These results indicate different relationship between *mesothelin* expression and gene mutations in colorectal and lung adenocarcinomas.

In normal tissues, mesothelin expression was detected in the mesothelial cells, epithelium of the fallopian tubes and seminal vesicles. Also, focal expression was seen in Hassall's corpuscles of the thymus and a subset of squamous epithelial cells of the tonsils. The biologic function of mesothelin is not well known including in these tissues.

In conclusion, many tumors such as mesothelioma and ovarian serous carcinoma showed equal immunoreactivity with both 5B2 and MN-1 antibodies, however, differences in mesothelin expression were seen in other tumor types using 5B2 or MN-1 antibodies. This has to be taken into account when selecting patients for anti-mesothelin immunotherapies. Mesothelin immunohistochemistry may also assist in the differential diagnosis of thymoma versus thymic carcinoma. Furthermore, mesothelin immunohistochemistry could be useful for the prognostication of malignant pleural mesothelioma patients.

## MATERIALS AND METHODS

### Tissue samples

This project was completed under Office of Human Subject Research Exemption with anonymized specimens. For the analysis of physiological expression, a panel of normal tissues was evaluated for mesothelin expression. For neoplastic conditions, 1562 of extensively characterized tumors were subjected to immunohistochemical analyses. Tumor samples derived from surgical specimens were assembled into multitumor blocks containing up to 60 rectangular tissue samples as previously described [24]. The size of tumor sample was estimated to exceed the size of a single 0.6mm^2^ core by a factor of 10–15.

### Antibodies and cells

The information of the antibodies has been summarized in Table 5. It was noted that 5B2 was generated by immunizing mice with a recombinant prokaryotic fusion protein corresponding to 100 amino acids which is also present in the N terminal Region I of mesothelin by the company (Novocastra/Leica, Bannockburn, IL). On the other hand, MN-1 was generated in mesothelin-deficient mice by immunization with plasmid cDNA corresponding to the mesothelin extracellular domain followed by a single boost with a recombinant human mesothelin-Fc fusion protein [22]. The validity of 5B2 and MN-1 antibodies was confirmed by immunoblot analyses using malignant pleural mesothelioma and pancreatic cancer cells. (Supplementary Figure 1) PANC-1 and SUIT-2 human pancreatic ductal adenocarcinoma cell lines were obtained from the RIKEN BioResource Center (Tsukuba, Japan). The human malignant pleural mesothelioma cell lines, ACC-MESO-4 and Y-MESO-8A, were kindly provided by Dr. Yoshitaka Sekido (Aichi Cancer Center Research Institute) [25].

**Table 5:** Antibodies and conditions for mesothelin immunohistochemistry

Antibody (Clone)	Retrieval	Dilution	
5B2	H2	100	Novocastra/Leica (Bannockburn, IL)
MN-1	H2	2,000	Rockland Immunochemicals Inc. (Limerick, PA)
MPF44	H1	1,000	Provided from collaborators, MO and IP
MPF25	H2	500	Provided from collaborators, MO and IP
MPH49	H2	500	Provided from collaborators, MO and IP

Antigen retrieval was performed with heat activation in Bond low (H1) or high (H2) pH buffer.

### Immunohistochemistry

Immunohistochemistry was performed using the Leica Bond-Max automation and Leica Refine detection kit (Leica Biosystems, Bannockburn, IL). The conditions for immunostaining have been summarized in Table 5. Signals were visualized by DAB. Mesothelin immunoreactivity (luminal/membranous or cytoplasmic) was evaluated with a detection cut-off of 5%. To compare the mesothelin positivity in small rectangular tissue samples on the tissue array and the corresponding larger whole-slide donor samples, 31 cases were selected for comparative immunohistochemical analysis. The results showing a high concordance are illustrated in Supplementary Figure 2. Immunohistochemistry of MLH1, MSH2, MSH6, and PMS2 were performed as previously reported [26, 27].

### Sequencing analysis

In colorectal tumors, arbitrary selected 75 samples were subjected to sequencing analysis in *BRAF*, *KRAS*, and *NRAS* genes. Mutation analyses were done by PCR and Sanger sequencing as previously reported [26]. Primer sequences, PCR conditions and size of amplicons are provided in Supplementary Table 4.

### Statistical analysis

All statistical analyses were performed with EZR version 1.32. software [28]. Chi-square or Fisher's exact test were performed to investigate the statistical correlation. To analyze the impact of the mesothelin expression on overall survival of mesothelioma patients, Kaplan-Meier survival estimates with log-rank test was performed. Cox proportional hazards regression analysis was used to analyze the association of survival and other factors. The initial model included age (< 65 vs. ≧ 65 years old), sex (male vs. female), tumor histology (epithelial vs. biphasic vs. sarcomatoid) and data from immunohistochemistry (diffuse mesothelin expression: 100% of positive cells on the cell membrane vs. negative or partial mesothelin expression in any location) for mesothelin with MN-1 and 5B2 antibody. A backward elimination with a threshold of *P* = 0.05 was used to select variables in the final model. Cases with missing information were eliminated from the statistical analysis of that parameter.

## SUPPLEMENTARY MATERIALS TABLES AND FIGURES



## References

[1] Chang K, Pastan I (1996). Molecular cloning of mesothelin, a differentiation antigen present on mesothelium, mesotheliomas, and ovarian cancers. Proc Natl Acad Sci USA.

[2] Bera TK, Pastan I (2000). Mesothelin is not required for normal mouse development or reproduction. Mol Cell Biol.

[3] Chang K, Pai LH, Pass H, Pogrebniak HW, Tsao MS, Pastan I, Willingham MC (1992). Monoclonal antibody K1 reacts with epithelial mesothelioma but not with lung adenocarcinoma. Am J Surg Pathol.

[4] Hassan R, Kreitman RJ, Pastan I, Willingham MC (2005). Localization of mesothelin in epithelial ovarian cancer. Appl Immunohistochem Mol Morphol.

[5] Hassan R, Laszik ZG, Lerner M, Raffeld M, Postier R, Brackett D (2005). Mesothelin is overexpressed in pancreaticobiliary adenocarcinomas but not in normal pancreas and chronic pancreatitis. Am J Clin Pathol.

[6] Miettinen M, Sarlomo-Rikala M (2003). Expression of calretinin, thrombomodulin, keratin 5, and mesothelin in lung carcinomas of different types: an immunohistochemical analysis of 596 tumors in comparison with epithelioid mesotheliomas of the pleura. Am J Surg Pathol.

[7] Ordonez NG (2003). Application of mesothelin immunostaining in tumor diagnosis. Am J Surg Pathol.

[8] Kachala SS, Bograd AJ, Villena-Vargas J, Suzuki K, Servais EL, Kadota K, Chou J, Sima CS, Vertes E, Rusch VW, Travis WD, Sadelain M, Adusumilli PS (2014). Mesothelin overexpression is a marker of tumor aggressiveness and is associated with reduced recurrence-free and overall survival in early-stage lung adenocarcinoma. Clin Cancer Res.

[9] Thomas A, Chen Y, Steinberg SM, Luo J, Pack S, Raffeld M, Abdullaev Z, Alewine C, Rajan A, Giaccone G, Pastan I, Miettinen M, Hassan R (2015). High mesothelin expression in advanced lung adenocarcinoma is associated with KRAS mutations and a poor prognosis. Oncotarget.

[10] Einama T, Homma S, Kamachi H, Kawamata F, Takahashi K, Takahashi N, Taniguchi M, Kamiyama T, Furukawa H, Matsuno Y, Tanaka S, Nishihara H, Taketomi A (2012). Luminal membrane expression of mesothelin is a prominent poor prognostic factor for gastric cancer. Br J Cancer.

[11] Gubbels JA, Belisle J, Onda M, Rancourt C, Migneault M, Ho M, Bera TK, Connor J, Sathyanarayana BK, Lee B, Pastan I, Patankar MS (2006). Mesothelin-MUC16 binding is a high affinity, N-glycan dependent interaction that facilitates peritoneal metastasis of ovarian tumors. Mol Cancer.

[12] Hassan R, Ebel W, Routhier EL, Patel R, Kline JB, Zhang J, Chao Q, Jacob S, Turchin H, Gibbs L, Phillips MD, Mudali S, Iacobuzio-Donahue C (2007). Preclinical evaluation of MORAb-009, a chimeric antibody targeting tumor-associated mesothelin. Cancer Immun.

[13] Kaneko O, Gong L, Zhang J, Hansen JK, Hassan R, Lee B, Ho M (2009). A binding domain on mesothelin for CA125/MUC16. J Biol Chem.

[14] Bharadwaj U, Marin-Muller C, Li M, Chen C, Yao Q (2011). Mesothelin overexpression promotes autocrine IL-6/sIL-6R trans-signaling to stimulate pancreatic cancer cell proliferation. Carcinogenesis.

[15] Chang MC, Chen CA, Hsieh CY, Lee CN, Su YN, Hu YH, Cheng WF (2009). Mesothelin inhibits paclitaxel-induced apoptosis through the PI3K pathway. Biochem J.

[16] Servais EL, Colovos C, Rodriguez L, Bograd AJ, Nitadori J, Sima C, Rusch VW, Sadelain M, Adusumilli PS (2012). Mesothelin overexpression promotes mesothelioma cell invasion and MMP-9 secretion in an orthotopic mouse model and in epithelioid pleural mesothelioma patients. Clin Cancer Res.

[17] Kelly RJ, Sharon E, Pastan I, Hassan R (2012). Mesothelin-targeted agents in clinical trials and in preclinical development. Mol Cancer Ther.

[18] Hassan R, Sharon E, Schuler B, Mallory Y, Zhang J, Ling A, Pastan I (2011). Antitumor activity of SS1P with pemetrexed and cisplatin for front-line treatment of pleural mesothelioma and utility of serum mesothelin as a marker of tumor response. J Clin Oncol.

[19] Hollevoet K, Nackaerts K, Gosselin R, De Wever W, Bosquee L, De Vuyst P, Germonpre PR, Kellen E, Legrand C, Kishi Y, Delanghe JR, Van Meerbeeck JP (2011). Soluble mesothelin, megakaryocyte potentiating factor, and osteopontin as markers of patient response and outcome in malignant pleural mesothelioma. J Clin Oncol.

[20] Linch M, Gennatas S, Kazikin S, Iqbal J, Gunapala R, Priest K, Severn J, Norton A, Ayite B, Bhosle J, O'Brien M, Popat S (2014). A serum mesothelin level is a prognostic indicator for patients with malignant mesothelioma in routine clinical practice. BMC Cancer.

[21] Zhang J, Khanna S, Jiang Q, Alewine C, Miettinen M, Pastan I, Hassan R (2016). Efficacy of Anti-Mesothelin Immunotoxin RG7787 plus nab-Paclitaxel against Mesothelioma Patient Derived Xenografts and Mesothelin as a Biomarker of Tumor Response. Clin Cancer Res.

[22] Onda M, Willingham M, Nagata S, Bera TK, Beers R, Ho M, Hassan R, Kreitman RJ, Pastan I (2005). New monoclonal antibodies to mesothelin useful for immunohistochemistry, fluorescence-activated cell sorting, Western blotting, and ELISA. Clin Cancer Res.

[23] Onda M, Nagata S, Ho M, Bera TK, Hassan R, Alexander RH, Pastan I (2006). Megakaryocyte potentiation factor cleaved from mesothelin precursor is a useful tumor marker in the serum of patients with mesothelioma. Clin Cancer Res.

[24] Miettinen M (2012). A simple method for generating multitissue blocks without special equipment. Appl Immunohistochem Mol Morphol.

[25] Usami N, Fukui T, Kondo M, Taniguchi T, Yokoyama T, Mori S, Yokoi K, Horio Y, Shimokata K, Sekido Y, Hida T (2006). Establishment and characterization of four malignant pleural mesothelioma cell lines from Japanese patients. Cancer Sci.

[26] Lasota J, Kowalik A, Wasag B, Wang ZF, Felisiak-Golabek A, Coates T, Kopczynski J, Gozdz S, Miettinen M (2014). Detection of the BRAF V600E mutation in colon carcinoma: critical evaluation of the imunohistochemical approach. Am J Surg Pathol.

[27] Inaguma S, Wang Z, Lasota J, Sarlomo-Rikala M, McCue PA, Ikeda H, Miettinen M (2016). Comprehensive Immunohistochemical Study of Programmed Cell Death Ligand 1 (PD-L1): Analysis in 5536 Cases Revealed Consistent Expression in Trophoblastic Tumors. Am J Surg Pathol.

[28] Kanda Y (2013). Investigation of the freely available easy-to-use software ‘EZR’ for medical statistics. Bone Marrow Transplant.

