# Supplementary material for: Comprehensive immunohistochemical study of mesothelin (MSLN) using different monoclonal antibodies 5B2 and MN-1 in 1562 tumors with evaluation of its prognostic value in malignant pleural mesothelioma
Source: Oncotarget. 2017 Mar 1;8(16):26744–54. doi: 10.18632/oncotarget.15814 (PMC5432294; doi:10.18632/oncotarget.15814)
Supplement: Supplementary file 1 [file oncotarget-08-26744-s001.pdf]

Comprehensive immunohistochemical study of mesothelin (MSLN) using different monoclonal antibodies 5B2 and MN-1 in 1562 tumors with evaluation of its prognostic value in malignant pleural mesothelioma

Supplementary Materials

Supplementary Table 1: Mismatch repair system-status in 77 gastric adenocarcinomas

MMR-status	Total No.		MN-1				*P*-value
			Luminal/membrane		Negative/cytoplasmic		
	77 [100%]	(100%)	37 [100%]	(48%)	40 [100%]	(52%)	
							0.08
Deficient	9 [12%]	(100%)	7 [19%]	(78%)	2 [5%]	(22%)	
Preserved	68 [88%]	(100%)	30 [81%]	(44%)	38 [95%]	(56%)	

Fisher's exact test.

Supplementary Table 2: Mismatch repair system-status in 183 colorectal adenocarcinomas

MMR-status	Total No.		MN-1				*P*-value
			Luminal/membrane		Negative/cytoplasmic		
	183 [100%]	(100%)	113 [100%]	(62%)	70 [100%]	(38%)	
							0.51
Deficient	29 [16%]	(100%)	20 [18%]	(69%)	9 [13%]	(31%)	
Preserved	154 [84%]	(100%)	93 [82%]	(60%)	61 [87%]	(40%)	

Chi-square test.

Supplementary Table 3: *BRAF* and *RAS* mutants in 75 colorectal adenocarcinomas

Gene mutation	Total No.		MN-1				*P*-value
			Luminal/membrane		Negative/cytoplasmic		
	75 [100%]	(100%)	50 [100%]	(67%)	25 [100%]	(33%)	
							0.82
BRAF mutant	20 [27%]	(100%)	13 [26%]	(65%)	7 [28%]	(35%)	
RAS mutant	31 [41%]	(100%)	22* [44%]	(71%)	9** [36%]	(29%)	
Wild type	24 [32%]	(100%)	15 [30%]	(63%)	9 [36%]	(38%)	

Fisher's exact test. *: all of them were *KRAS* mutants, **: 8 *KRAS* and 1 *NRAS* mutants were included.

Supplementary Table 4: Primer sequences and PCR conditions used to amplify targets for sanger sequencing

Gene	Target	Forward primer (5′_3′)	Reverse primer (5′_3′)	A_temp (°C)	Amplicon (bp)
BRAF	Exon 15	TCTTCATGAAGACCTCACAG	AGCCTCAATTCTTACCATCC	50	125
KRAS	Exon 2	GTGTGACATGTTCTAATATAGTCA	AGAATGGTCCTGCACCAGAATTAT	48	215
KRAS	Exon 3	CCAGACTGTGTTTCTCCCTT	TACACAAAGAAAGCCCTCCC	50	157
KRAS	Exon 4	AGAGTTAAGGACTCTGAAGA	CAGTGTTACTTACCTGTCTT	45	220
NRAS	Exon 2	GACTGAGTACAAACTGGTGG	CACCTCTATGGTGGGATCAT	48	112
NRAS	Exon 3	CCCCCAGGATTCTTACAGAA	ATACACAGAGGAAGCCTTCG	49	140

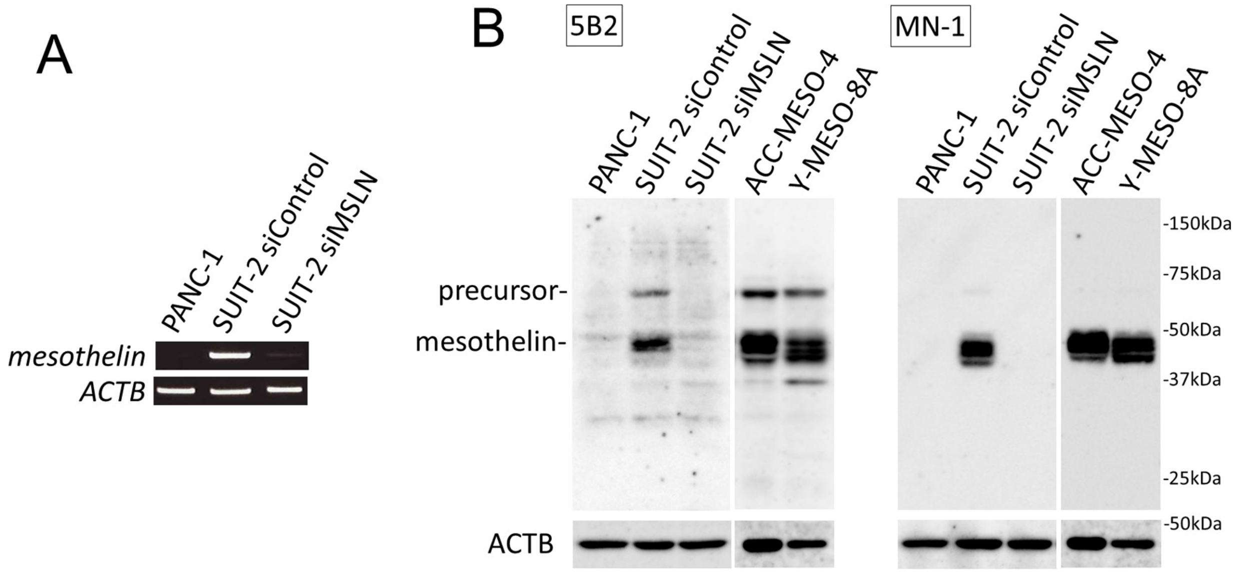

Supplementary Figure 1: Validation of 5B2 and MN-1 anti-mesothelin antibodies. (**A** and **B**), RT-PCR (A) and immunoblot (B) analyses of pancreatic cancer and malignant pleural mesothelioma cell lines. The 21-nucleotide duplex siRNAs were synthesized as follows: siMSLN, 5′-CCCGUUUCUUCUCCCGCAUTT-3′ and 5′-AUGCGGGAGAAGAAACGGGTT-3′; siControl, 5′-GACAACGACGAAAGAUACUTT-3′ and 5′-AGUAUCUUUCGUCGUUGUCTT-3′. After 48 hours of siRNA transfection, whole-cell lysate and total RNA were purified and subjected for RT-PCR and immunoblot analyses, respectively. For immunoblot analyses, the antibodies were applied at a dilution of 1:500 (5B2) and 1:40,000 (MN-1). Mesothelin precursor protein (71 kDa) and mesothelin (40 kDa) were detected in immunoblot analyses. However, MPF (megakaryocyte potentiating factor, 31 kDa) was not detected in both antibodies.

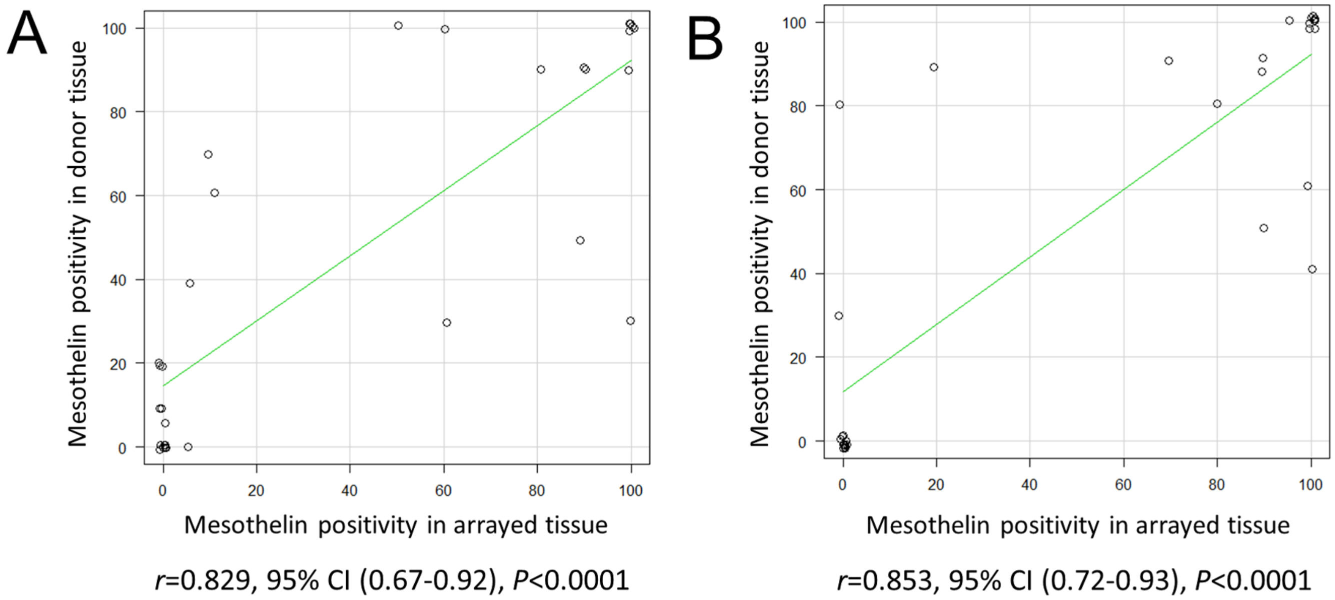

Supplementary Figure 2: Correlation of mesothelin positivity between arrayed tissue and donor whole sections. (**A** and **B**) correlations of the positivity stained with 5B2 (A) and MN-1 (B) antibodies. Selected 31 cases (ovary serous carcinoma, 4 cases; pancreas invasive ductal carcinoma, 3 cases; urothelial carcinoma, 4 cases; thymic carcinoma, 2 cases; thymoma, 3 cases; uterine endometrioid adenocarcinoma, 4 cases; lung squamous cell carcinoma, 4 cases; lung adenocarcinoma, 4 cases; colorectal adenocarcinoma, 3 cases) were compared for mesothelin expression in small samples on the tissue array and donor whole sections. For the statistical analyses, Pearson's correlation coefficient was used.
